# Clinical impact of tumor location on the colon cancer survival and recurrence: analyses of pooled data from three large phase III randomized clinical trials

**DOI:** 10.1002/cam4.1208

**Published:** 2017-09-25

**Authors:** Toru Aoyama, Kosuke Kashiwabara, Koji Oba, Michitaka Honda, Sotaro Sadahiro, Chikuma Hamada, Hiromichi Maeda, Shuhei Mayanagi, Mitsuro Kanda, Junichi Sakamoto, Shigetoyo Saji, Takaki Yoshikawa

**Affiliations:** ^1^ Department of Surgery Yokohama City University Yokohama Japan; ^2^ Department of Biostatistics The University of Tokyo Tokyo Japan; ^3^ Department of Minimally Invasive Surgical and Medical Oncology Fukushima Medical University Fukushima Japan; ^4^ Department of Surgery Tokai University Isehara Japan; ^5^ Faculty of Engineering Tokyo University of Science Tokyo Japan; ^6^ Cancer Treatment Center Kochi Medical School Hospital Kochi Japan; ^7^ Department of Surgery Keio University School of Medicine Tokyo Japan; ^8^ Department of Gastroenterological Surgery Nagoya University Graduate School of Medicine Nagoya Japan; ^9^ Tokai Central Hospital Kakamigahara Japan; ^10^ Japanese Foundation for Multidisciplinary Treatment of Cancer Tokyo Japan; ^11^ Department of Gastrointestinal Surgery Kanagawa Cancer Center Yokohama Kanagawa Japan

**Keywords:** Colorectal cancer, left side colon cancer, recurrence, right side colon cancer, survival

## Abstract

The aim of the present study was to determine whether or not the overall survival (OS) and disease‐free survival (DFS) were affected by the tumor location in patients who underwent curative resection for colon cancer in a pooled analysis of three large phase III studies performed in Japan. In total, 4029 patients were included in the present study. Patients were classified as having right‐side colon cancer (RC) if the primary tumor was located in the cecum, ascending colon, hepatic flexure or transverse colon, and left‐side colon cancer (LCC) if the tumor site was within the splenic flexure, descending colon, sigmoid colon or recto sigmoid junction. The risk factors for the OS and DFS were analyzed. In the present study, 1449 patients were RC, and 2580 were LCC. The OS rates at 3 and 5 years after surgery were 87.6% and 81.6% in the RC group and 91.5% and 84.5% in the LCC group, respectively. Uni‐ and multivariate analyses showed that RRC increased the risk of death by 19.7% (adjusted hazard ratio = 1.197; 95% confidence interval, 1.020–1.408; *P* = 0.0272). In contrast, the DFS was similar between the two locations. The present study confirmed that the tumor location was a risk factor for the OS in patients who underwent curative treatment for colon cancer. Tumor location may, therefore, need to be considered a stratification factor in future phase III trials of colon cancer.

## Introduction

Colorectal cancer is the third most commonly diagnosed cancer in males and the second most in females, with an estimated 1.4 million new cases and 693,900 deaths occurring in 2012 1. Complete resection is essential for a cure; however, while the resection rate has gradually increased, some patients experience recurrence even after curative surgery. Once recurrence has developed, the prognosis is poor 2, 3, 4. Therefore, it is important to determine reliable risk factors in order to identify patients at high risk of recurrence.

Bufill et al. originally proposed that right‐side colon cancer (RC) and left‐side colon cancer (LCC) might present via distinct biological pathways 5. It has been known for many years that RC and LCC represent distinct entities, with differences in epidemiology, biology, pathology, and clinical outcomes. Recently, the association between the tumor location and the prognosis has been explored in the metastatic setting 6, 7, 8. However, these studies focused on the treatment effect or response to chemotherapy, especially in terms of molecular targeting agents. Therefore, whether or not the tumor location can function as an important additional risk factor for patients and clinicians in daily practice and as a relevant stratification factor for clinical trials in an adjuvant setting remains unclear. Furthermore, most previous studies have evaluated retrospectively collected data from relatively small sample sizes from a single institution. Retrospective studies have many limitations, such as unspecified indications of surgery, heterogeneous populations, and heterogeneous treatments. To overcome these limitations associated with retrospective studies, we focused on cases that had been enrolled in large, randomized clinical trials of adjuvant chemotherapy by pooling individual patients' data 9, 10, 11.

The aim of the present study was to determine whether or not the overall survival (OS) and disease‐free survival (DFS) were affected by the tumor location in patients who underwent curative resection for colon cancer in a pooled analysis of three large phase III studies performed in Japan. These findings should help confirm the influence of the tumor location on the survival of colon cancer patients scheduled to undergo curative surgical resection.

## Patients and Methods

### Patients

The data and outcomes of patients enrolled in three phase III trials of Japanese Foundation for Multidisciplinary Treatment of Cancer (JFMC) studies (7, 15, and 33) were pooled 9, 10, 11.

### JFMC trials in the pooled analysis

#### JFMC 7 and 15

These two randomized trials were relatively large‐scale trials conducted in Japan that focused on the long‐term utilization of oral 5‐fluorouracil (FU) as adjuvant chemotherapy for colon or rectal cancer and compared the OS with the surgery‐alone arm or with one of the treatment arms. The basic study designs of these trials were very similar and included the following key eligibility criteria: stage of cancer, stage II, and III using AJCC/UICC 12 (originally described as macroscopic Dukes' B and C); age, <75 years; no severe complications; follow‐up period, 5 years. The main adjuvant chemotherapy was the 1‐year administration of oral 5‐FUs (JFMC7‐1: 200 mg/day 5‐FU; JFMC7‐2 and JFMC15:300 mg/day 1‐hexycarbamoyl‐5‐fluorouracil [carmofur, HCFU]). HCFU is an oral fluorinated pyrimidine developed as a 5‐FU lipophilic masked compound. All eligible patients were the target analysis set for the statistical analysis, totaling 3394 for the JFMC 7 trial between February 1986 and December 1988 and 2315 for the JFMC 15 trial between January 1989 and December 1990. The details of the protocol and primary analyses of JFMC 7 and 15 were reported previously.

#### Jfmc 33

This phase III trial randomly assigned 1071 eligible patients from 2005 to 2007 at 233 centers to receive tegafur (UFT, 300 mg/m^2^ per day as tegafur)/leucovorin (LV, 75 mg/day) for 28 of 35 days for 6 months in the control group or for five consecutive days per week for 18 months. Patients with curatively resected stage IIB/III colon cancer were eligible for enrollment in this trial. The primary endpoint was the OS. The details and primary analyses of JFMC 33 were reported previously.

In our pooled analysis, the eligible patients were those who had stage I/II/III colorectal cancer and underwent over D2 lymph node dissection. Thus, 754 and 496 patients were excluded from the intention‐to‐treat (ITT) cohort of the JFMC 7 and 15 studies, respectively. In total, 4029 patients were included in the present analysis. Among them, 846 patients received 5‐FU, 845 patients received 1‐hexycarbamoyl‐5‐fluorouracil, and 1070 patients received tegafur and leucovorin as adjuvant therapy. According to the NCCN guideline for stage III and high‐risk stage II (UICC classification) colon cancer was recommended to receive the 5‐FU based adjuvant treatment after curative surgery. Considering these, 68.5% of the patients were patients received the appropriate and recommended adjuvant therapy in the present study.

### RC versus LCC

Patients were classified as having RC if the primary tumor was located in the cecum, ascending colon, hepatic flexure, or transverse colon, and LCC if the tumor site was within the splenic flexure, descending colon, sigmoid colon, or recto sigmoid junction. Patients with primary rectal tumors and those whose primary tumor site could not be determined based on the provided information were excluded.

### Follow‐up

Patients were followed‐up according to the protocol of each study. Briefly, during protocol treatment, clinical findings, and laboratory data were evaluated every two weeks. After completion of the protocol treatment, patients were followed‐up according to a predefined surveillance schedule until recurrence or death was confirmed for 5 years after surgery. Recurrence was assessed based on computed tomography (CT) scans. These tests were carried out every 4 months during the first 2 years after surgery and once every 6 months from the third year onward.

### Evaluations and statistical analyses

The background characteristics of postoperative clinical and pathological parameters between the RC group and the LCC group were compared using the Mann–Whitney test or *χ* ^2^ test. The OS was defined as the period between surgery and any cause of death. The DFS was defined as the period between surgery and the occurrence of recurrence, second cancer, or death, whichever came first. The data for patients who had not experienced an event were censored as of the date of the final observation. The OS and DFS curves were calculated using the Kaplan–Meier method and compared by the log‐rank test. A Cox proportional hazards model was used to perform the univariate and multivariate survival analyses. In the multivariate analysis, adjuvant chemotherapy, age, gender, tumor diameter, tumor location, T category, lymph node metastases, stage, lymphadenectomy, and histology were considered as confounders based on a priori knowledge. A value of two‐sided *P* < 0.05 was defined as being statistically significant. The SAS version 9.4 software program (SAS Institute Inc., Cary, NC) was used for all statistical analyses. We revised and transformed to current staging terminology using AJCC/UICC TNM staging system 12. Moreover, in the present study, the histological parameters were evaluated on H&E‐stained slides by pathologists of each institution in a blinded manner. Mucinous adenocarcinoma is diagnosed as colon cancer in which >50% of the tumor is composed of mucin. Poorly differentiated adenocarcinoma is diagnosed according to the World Health Organization (2000) classification as tumors with glandular structures comprising 5–50% of the tumor 13, 14, 15. This study was approved by the ethics committee of the Japanese Foundation for Multidisciplinary Treatment of Cancer.

## Results

### Patients

The data of 4029 individual patients were evaluated in this study. The patients' ages ranged from 23 to 75 years (median: 61 years); 2176 patients were male, and 1853 were female. The median follow‐up period was 5 years. Patients' demographics and clinical characteristics are summarized (Table 1). On comparing RC and LCC, there were significant differences observed in age, gender, tumor diameter, UICC T category, lymph node dissection, and histology status. Patients with RC were significantly older, predominantly women, and had a larger tumor diameter and a higher proportion of poorly differentiated type and mucinous type disease than those with LCC.

**Table 1:** Comparison clinicopathological factors between the patients with right‐side colon cancer group and the patients with left‐side colon cancer group

Factors	All cases (*n* = 4029)		Right‐side colon cancer group (*n* = 1449)		Left‐side colon cancer group (*n* = 2580)		*P*‐value
	Number	(%)	Number	(%)	Number	(%)	
Gender							<0.0001
Male	2176	54.0	706	48.7	1470	57.0	
Female	1853	46.0	743	51.3	1110	43.0	
Age (years)							<0.0001
Median	61 years (23–75)		62 years (23–75)		60 years (24–75)		
Diameter of tumor (mm)							<0.0001
Median	50 mm (10–280)		55 mm (10–155)		49 mm (10–280)		
Histology							<0.0001
Well‐moderately differentiated	3795	94.2	1293	89.2	2502	97.0	
Poorly differentiated	177	2.9	82	5.7	35	1.4	
Muc	117	2.9	74	5.1	43	1.7	
UICC T status							0.0077
T1–T3	2912	72.3	1008	69.6	1904	73.8	
T4	1102	27.4	433	29.9	669	25.9	
Missing	15	0.3	8	0.5	7	0.3	
LN node metastases							0.2587
Negative	2157	53.5	762	52.6	1395	54.1	
Positive	1857	46.1	679	46.9	1178	45.6	
Missing	15	0.4	8	0.5	7	0.3	
Lymph node dissection							0.0009
D2	1526	37.9	500	34.5	1026	39.8	
D3	2497	62.0	945	65.2	1552	60.1	
Missing	6	0.1	4	0.3	2	0.1	
Adjuvant chemotherapy							0.0899
Yes	2761	68.5	1017	70.2	1744	67.6	
No	1268	31.5	432	29.8	836	32.4	

UICC, Union for International Cancer Control; LN, lymph node.

### Survival analyses

**Figure 1:**
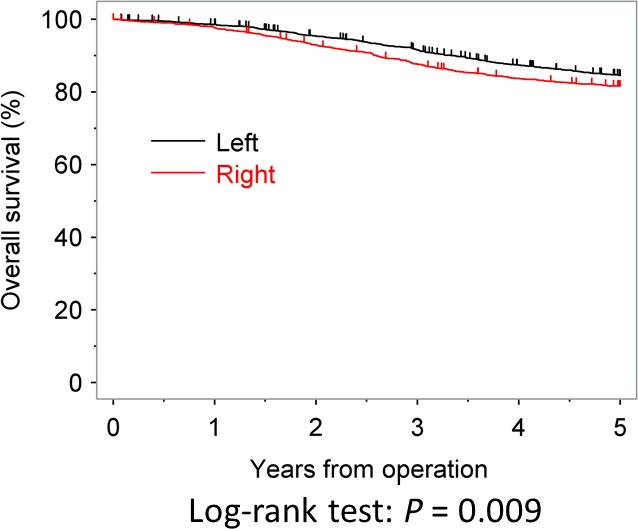
The overall survival curves of right‐side colon cancers and left‐side colon cancers.

The OS rates at 3 and 5 years after surgery were 87.6% and 81.6% in the RC group and 91.5% and 84.5% in the LCC group, respectively; these values were significantly different between the two groups (*P* < 0.009). The OS curves are shown in Figure 1. In the univariate analysis, age, postoperative complication, gender, tumor diameter, tumor location, the T category, N category, lymphadenectomy, and use of adjuvant chemotherapy were all found to be significantly associated with the OS. The multivariate analysis showed that RC increased the risk of death by 19.7% (adjusted hazard ratio (HR) = 1.197; 95% confidence interval (CI), 1.020–1.405; *P* = 0.0272; Table 2).

**Table 2:** Uni and Multivariate Cox proportional hazards analysis of clinicopathological factors for overall survival

Factors	Number	Univariate analysis			Multivariate analysis		
		OR	95% CI	*P* value	OR	95% CI	*P* value
Age (years)				0.0068			0.0031
–<60	1785	1.000			1.000		
60≦–	2244	1.241	1.061–1.451		1.271	1.084–1.491	
Gender				0.1209			0.1216
Female	1853	1.000			1.000		
Male	2176	1.130	0.968–1.319		1.132	0.968–1.323	
Tumor diameter (mm)				0.0180			0.0272
–<50	1811	1.000			1.000		
50≦–	2211	1.207	1.033–1.410		1.199	1.020–1.408	
Tumor location				0.0124			0.0272
Left side colon	2580	1.000			1.000		
Right side colon	1449	1.220	1.044–1.427		1.197	1.020–1.405	
UICC T category				<0.001			<0.001
T1–T3	2912	1.000			1.000		
T4	1102	1.589	1.355–1.863		1.578	1.337–1.861	
Lymph node metastases				<0.001			<0.001
Negative	2157	1.000			1.000		
Positive	1857	1.970	1.683–2.305		2.520	2.128–2.983	
Surgical Complication				0.0117			0.0206
No	3610	1.000			1.000		
Yes	419	1.341	1.067–1.684		1.313	1.043–1.653	
Lymph node dissection				0.0074			0.0133
D3	2497	1.000			1.000		
D2	1526	1.236	1.058–1.442		1.220	1.042–1.429	
Adjuvant treatment							
Surgery alone	1268	1.000			1.000		
5‐FU	846	0.890	0.736–1.075	0.2256	0.883	0.714–1.092	0.2518
HCFU	845	0.881	0.728–1.065	0.1905	1.005	0.809–1.248	0.9639
UFT/LV	1070	0.822	0.696–0.969	0.0203	0.526	0.422–0.656	<0.001

UICC, Union for International Cancer Control; 5‐FU, 5‐fluorouracil; HCFU, 1‐hexylcarbamoyl‐5‐fluorouracil; UFT/LV, Tegafur/Uracil/leucovorin.

**Figure 2:**
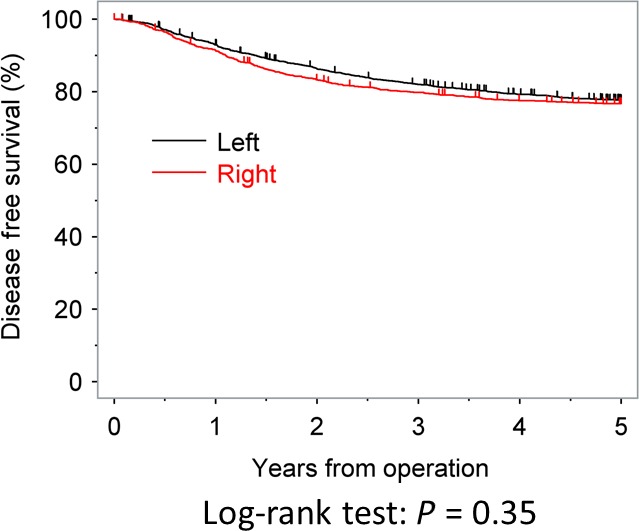
The recurrence‐free survival curves of right‐side colon cancers and left‐side colon cancers.

The DFS rates at 3 and 5 years after surgery were 79.8% and 76.7% in the RC group and 82.0% and 77.6% in the LCC group, respectively. The DFS was similar between the two groups (*P* = 0.3500). The DFS curves are shown in Figure 2. In contrast to the OS, the tumor location was not found to be significantly associated with the DFS in either uni‐ or multivariate analyses (Table 3).

**Table 3:** Uni and Multivariate Cox proportional hazards analysis of clinicopathological factors for recurrence free survival

Factors	Number	Univariate analysis			Multivariate analysis		
		OR	95% CI	*P* value	OR	95% CI	*P* value
Age (years)				0.0140			0.0385
–<60	1785	1.000			1.000		
60≦–	2244	1.181	1.034–1.348		1.154	1.008–1.321	
Gender				0.0095			0.0069
Female	1853	1.000			1.000		
Male	2176	1.191	1.044–1.359		1.202	1.052–1.374	
Tumor diameter (mm)				0.6115			0.2107
–<50	1811	1.000			1.000		
50≦–	2211	1.035	0.907–1.180		1.091	0.952–1.250	
Tumor location				0.3734			0.4919
Left side colon	2580	1.000			1.000		
Right side colon	1449	1.063	0.929–1.217		1.050	0.914–1.205	
UICC T category				<0.001			<0.001
T1–T3	2912	1.000			1.000		
T4	1102	1.616	1.411–1.850		1.605	1.395–1.848	
Lymph node metastases				<0.001			<0.001
Negative	2157	1.000			1.000		
Positive	1857	2.249	1.964–2.575		2.462	2.124–2.853	
Surgical Complication				0.0260			0.0159
No	3610	1.000			1.000		
Yes	419	1.254	1.027–1.531		1.282	1.047–1.568	
Lymph node dissection				0.0346			0.0089
D3	2497	1.000			1.000		
D2	1526	1.154	1.010–1.317		1.198	1.046–1.371	
Adjuvant chemotherapy							
Surgery alone	1268	1.000			1.000		
5‐FU	846	0.925	0.748–1.143	0.4688	0.845	0.699–1.022	0.0833
HCFU	845	0.928	0.750–1.148	0.4889	0.967	0.797–1.174	0.7367
UFT/LV	1070	0.822	0.670–1.008	0.0597	0.799	0.667–0.956	0.0143

UICC, Union for International Cancer Control; 5‐FU, 5‐fluorouracil; HCFU, 1‐hexylcarbamoyl‐5‐fluorouracil; UFT/LV, Tegafur/Uracil/leucovorin.

### Subgroup analysis

Subgroup analyses were performed according to the UICC T stage and N stage. In patients who were T1–T3, the adjusted hazard ratio of the tumor location for the OS and DFS was 1.049 (95% CI, 0.853–1.289) and 0.951 (95% CI, 0.796–1.136), respectively. In patients who were T4, the adjusted hazard ratio of the tumor location for the OS and DFS was 1.405 (95% CI, 1.079–1.830) and 1.204 (95% CI, 0.960–1.510), respectively. In patients who were lymph node metastasis‐negative, the adjusted hazard ratio of the tumor location for the OS and DFS was 1.179 (95% CI, 0.909–1.529) and 1.159 (95% CI, 0.917–1.465), respectively. In patients were lymph node metastasis positive, the adjusted hazard ratio of the tumor location for the OS and DFS was 1.196 (95% CI, 0.972–1.472) and 1.067 (95% CI, 0.896–1.272), respectively.

Subgroup analyses were also performed according to the type of treatment after surgery. In patients randomized to the surgery‐alone group, the adjusted hazard ratio of the tumor location for the OS and DFS was 1.035 (95% CI, 0.777–1.378) and 0.864 (95% CI, 0.666–1.120), respectively. In patients randomized to the adjuvant chemotherapy group, the adjusted hazard ratio of the tumor location for the OS and DFS was 1.243 (95% CI, 1.022–1.513) and 1.126 (95% CI, 0.955–1.329), respectively. Moreover, the adjusted hazard ratio of the tumor location for the OS and DFS was 1.323 (95% CI, 0.936–1.869) and 1.341 (95% CI, 0.960–1.798) in 5‐FU, 0.874 (95% CI, 0.599–1.274) and 0.799 (95% CI, 0.567–1.126) in HCFU, and 1.645 (95% CI, 1.193–2.270) and 1.270 (95% CI, 0.996–1.619) in UFT/LV, respectively.

## Discussion

The present study examined whether or not the tumor location was associated with a poorer OS and DFS after curative surgery for colon cancer in combined analyses of individual patients' data from the three large phase III studies evaluating the effects of adjuvant treatment. Our findings clearly indicated that the tumor location was a significant independent risk factor for the OS. Furthermore, we found a significant interaction between the tumor location and the OS in patients who had received adjuvant chemotherapy.

In the present study, the adjusted hazard ratio for the OS in RC compared to LCC was 1.197 (95% CI, 1.020–1.405). A similar hazard ratio and 95% CI were observed in previous large observational studies. Robert et al. examined 77978 colon cancer patients and found that RC was associated with a worse prognosis than LCC 16. The median survival for RC was 78 versus 89 months for LCC. In addition, RC was associated with a 5% increased mortality risk compared with LCC (HR, 1.04; 95% CI, 1.02–1.07). Benedix et al. examined 17641 colon cancer patients and found that the 5‐year OS rate was higher for patients with LCC (RC 67% vs. LCC 71%) 17. They also reported that the hazard ratio was 1.120 (95% CI, 1.018–1.226). The reason for the observed difference in the survival for subjects with RC versus LCC is unclear. One possible reason might be that RC is found more frequently in older people and is more likely to be poorly differentiated and mucinous 18, 19. These findings of characteristic differences between RC and LCC were also observed in the present study.

Another possible reason might be due to differences in the molecular biologic pattern between RC and LCC. RC and LCC show huge differences in their molecular and cellular features. Microsatellite instability (MSI) is more common on the right side. MSI may not only have “general” prognostic implications, it may also explain the interrelationship with treatment and prognosis and location 20. In addition, BRAF mutations are more common on the right side, which may also have affected the prognosis 21. On the other hands, LCC often shows chromosomal instability 22, 23, 24. Furthermore, recent studies have shown that LCC benefits more from cetuximab treatment than RC among KRAS wild‐type cancers 25. In addition, advanced LCC shows a higher sensitivity to bevacizumab treatment than advanced RC 26, 27. The differences in the molecular biologic pattern, which affect the efficacy of treatment after recurrence, might affect the OS. However, we did not perform genomic analysis in the present study. Moreover, there is no inclusion of patients getting epidermal growth factor receptor or vascular endothelial growth factor inhibition in the patients who registered to the JFMC 7 and 15. On the other hands, there might be inclusion of patients getting epidermal growth factor receptor or vascular endothelial growth factor inhibition after recurrence in the patients who registered to the JFMC 33. However, we did not collect this information. Therefore, the present study could not show the relations and impacts between genomic difference and tumor location.

In contrast, the DFS did not differ significantly between RC and LCC. Similar trends were observed in previous reports 16, 17. Why there is an obvious difference between the OS and DFS between these two groups remains unclear and must be explored in future studies.

In the subgroup analyses, when the OS and DFS were analyzed with respect to the UICC T stage and N stage, there was no significant difference in either the OS or DFS. This is also consistent with the recently published results 16, 17. However, a significant interaction was observed between the tumor location and the OS in patients who had received adjuvant chemotherapy. In addition, marginal interactions were observed between the tumor location and the OS in each regimen. Therefore, the tumor location might influence the efficacy of adjuvant chemotherapy treatment, especially in patients receiving 5‐FU‐based chemotherapy. In contrast, although the OS was slightly worse in RC patients than in LCC patients, no significant interaction was observed between the tumor location and the OS in patients treated only with surgery. However, only 1268 patients received surgery alone. These marginal differences might become more important if the number of patients is increased.

The present study had much strength such as huge well‐defined cohorts, long follow‐up and individual patient's data. Moreover, 68.5% of the patients were patients received the appropriate and recommended adjuvant therapy in the present study. However, several limitations warrant mention. First, the patients in this cohort met the inclusion criteria of each clinical trial, which may have contained selection bias. Second, there was a time bias in this study, as the data were collected over a relatively long period from 1986 to 2007. The details of chemotherapies might have changed over such a long span of time. A further important limitation associated with all of the available data regarding tumor location, including the current study, is the lack of consensus regarding the appropriate cut‐off point of the tumor location. In the present study, RC and LCC were not defined in the protocols of the three phase III studies; instead, RC and LCC were reported by the individual physicians and were not based on a specific protocol. This will likely strongly affect the utility of the tumor location as stratification factor in future clinical trials.

In conclusion, the present study confirmed that the tumor location was a risk factor for the OS in patients who underwent curative surgery for colon cancer. Tumor location may, therefore, be considered as a stratification factor in future phase III trials of colon cancer.

## Conflict of Interest

None declared.

## References

[1] Torre, L. A. , F. Bray , R. L. Siegel , J. Ferlay , J. Lortet‐Tieulent , and A. Jemal . 2015 Global cancer statistics, 2012. CA Cancer J. Clin. 65:87–108. 25651787 10.3322/caac.21262

[2] Douillard, J. Y. , D. Cunningham , A. D. Roth , M. Navarro , R. D. James , P. Karasek , et al. 2000 Irinotecan combined with fluorouracil compared with fluorouracil alone as first‐line treatment for metastatic colorectal cancer: a multicentre randomised trial. Lancet 355:1041–1047. 10744089 10.1016/s0140-6736(00)02034-1

[3] Saltz, L. B. , J. V. Cox , C. Blanke , L. S. Rosen , L. Fehrenbacher , M. J. Moore , et al. 2000 Irinotecan plus fluorouracil and leucovorin for metastatic colorectal cancer. Irinotecan Study Group. N. Engl. J. Med. 343:905–914. 11006366 10.1056/NEJM200009283431302

[4] Köhne, C. H. , E. van Cutsem , J. Wils , C. Bokemeyer , M. El‐Serafi , M. P. Lutz , et al. 2005 Phase III study of weekly high‐dose infusional fluorouracil plus folinic acid with or without irinotecan in patients with metastatic colorectal cancer: European Organisation for Research and Treatment of Cancer Gastrointestinal Group Study 40986. J. Clin. Oncol. 23:4856–4865. 15939923 10.1200/JCO.2005.05.546

[5] Bufill, J. A. 1990 Colorectal cancer: evidence for distinct genetic categories based on proximal or distal tumor location. Ann. Intern. Med. 113:779–788. 2240880 10.7326/0003-4819-113-10-779

[6] Modest, D. P. , C. Schulz , L. F. von Weikersthal , D. Quietzsch , J. C. von Einem , A. Schalhorn , et al. 2014 Outcome of patients with metastatic colorectal cancer depends on the primary tumor site (midgut vs. hindgut): analysis of the FIRE1‐trial (FuFIRI or mIROX as first‐line treatment). Anticancer Drugs. 25:212–218. 24201305 10.1097/CAD.0000000000000041

[7] von Einem, J. C. , V. Heinemann , L. F. von Weikersthal , U. Vehling‐Kaiser , M. Stauch , H. G. Hass , et al. 2014 Left‐sided primary tumors are associated with favorable prognosis in patients with KRAS codon 12/13 wild‐type metastatic colorectal cancer treated with cetuximab plus chemotherapy: an analysis of the AIO KRK‐0104 trial. J. Cancer Res. Clin. Oncol. 140:1607–1614. 24816724 10.1007/s00432-014-1678-3 PMC4131148

[8] Brulé, S. Y. , D. J. Jonker , C. S. Karapetis , C. J. O'Callaghan , M. J. Moore , R. Wong , et al. 2015 Location of colon cancer (right‐sided versus left‐sided) as a prognostic factor and a predictor of benefit from cetuximab in NCIC CO.17. Eur. J. Cancer. 51:1405–1414. 25979833 10.1016/j.ejca.2015.03.015

[9] Hamada, C. , J. Sakamoto , T. Satoh , S. Sadahiro , H. Mishima , K. Sugihara , et al. 2011 Does 1 year adjuvant chemotherapy with oral 5‐FUs in colon cancer reduce the peak of recurrence in 1 year and provide long‐term OS benefit? Jpn. J. Clin. Oncol. 41:299–302. 21084437 10.1093/jjco/hyq209

[10] Sadahiro, S. , T. Tsuchiya , K. Sasaki , K. Kondo , K. Katsumata , G. Nishimura , et al. 2015 Randomized phase III trial of treatment duration for oral uracil and tegafur plus leucovorin as adjuvant chemotherapy for patients with stage IIB/III colon cancer: final results of JFMC33‐0502. Ann. Oncol. 26:2274–2280. 26347106 10.1093/annonc/mdv358 PMC4621030

[11] Aoyama, T. , K. Oba , C. Hamada , S. Sadahiro , M. Honda , S. Mayanagi , et al. 2016 Comparison of clinic pathological characters and survival between right‐ and left‐side colon cancer. Ann. Cancer Res. Ther. 24:62–63.

[12] Edge S. B., Byrd D. R., Compton C. C., Fritz A. G., Greene F. L., and Trotti A., eds. 2010 AJCC cancer staging manual, 7th ed Springer, New York.

[13] Brenner, H. , M. Kloor , and C. P. Pox . 2014 Colorectal cancer. Lancet 383:1490–1502. 24225001 10.1016/S0140-6736(13)61649-9

[14] Park, J. S. , J. W. Huh , Y. A. Park , et al. 2015 Prognostic comparison between mucinous and nonmucinous adenocarcinoma in colorectal cancer. Medicine (Baltimore). 94:e658. 25881840 10.1097/MD.0000000000000658 PMC4602499

[15] Kazama, Y. , T. Watanabe , T. Kanazawa , et al. 2007 Microsatellite instability in poorly differentiated adenocarcinomas of the colon and rectum: relationship to clinicopathological features. J. Clin. Pathol. 60:701–704. 17557871 10.1136/jcp.2006.039081 PMC1955052

[16] Meguid, R. A. , M. B. Slidell , C. L. Wolfgang , D. C. Chang , and N. Ahuja . 2008 Is there a difference in survival between right‐ versus left‐sided colon cancers? Ann. Surg. Oncol. 15:2388–2394. 18622647 10.1245/s10434-008-0015-y PMC3072702

[17] Benedix, F. , R. Kube , F. Meyer , U. Schmidt , I. Gastinger , and H. Lippert . 2010 Colon/Rectum Carcinomas (Primary Tumor) Study Group. Comparison of 17,641 patients with right‐ and left‐sided colon cancer: differences in epidemiology, perioperative course, histology, and survival. Dis. Colon Rectum. 53:57–64. 20010352 10.1007/DCR.0b013e3181c703a4

[18] Iacopetta, B. 2002 Are there two sides to colorectal cancer? Int. J. Cancer. 101:403–408. 12216066 10.1002/ijc.10635

[19] Nawa, T. , J. Kato , H. Kawamoto , H. Okada , H. Yamamoto , H. Kohno , et al. 2008 Differences between rightand left‐sided colon cancer in patient characteristics, cancer morphology and histology. J. Gastroenterol. Hepatol. 23:418–423. 17532785 10.1111/j.1440-1746.2007.04923.x

[20] Sugai, T. , W. Habano , Y. F. Jiao , M. Tsukahara , Y. Takeda , K. Otsuka , et al. 2006 Analysis of molecular alterations in left‐ and right‐sided colorectal carcinomas reveals distinct pathways of carcinogenesis: proposal for new molecular profile of colorectal carcinomas. J. Mol. Diagn. 8:193–201. 16645205 10.2353/jmoldx.2006.050052 PMC1867579

[21] Yokota, T. , T. Ura , N. Shibata , D. Takahari , K. Shitara , M. Nomura , et al. 2011 BRAF mutation is a powerful prognostic factor in advanced and recurrent colorectal cancer. Br. J. Cancer. 104:856–62. 21285991 10.1038/bjc.2011.19 PMC3048210

[22] Gervaz, P. , P. Bucher , and P. Morel . 2004 Two colons—two cancers: paradigm shift and clinical implications. J. Surg. Oncol. 88:261–6. 15565587 10.1002/jso.20156

[23] Popat, S. , R. Hubner , and R. S. Houlston . 2005 Systematic review of microsatellite instability and colorectal cancer prognosis. J. Clin. Oncol. 23:609–618. 15659508 10.1200/JCO.2005.01.086

[24] Malesci, A. , L. Laghi , P. Bianchi , et al. 2007 Reduced likelihood of metastases in patients with microsatellite‐unstable colorectal cancer. Clin. Cancer Res. 13:3831–3839. 17606714 10.1158/1078-0432.CCR-07-0366

[25] Missiaglia, E. , B. Jacobs , A. F. Di Narzo , C. Soneson , A. Roth , F. Bosman , et al. 2013 Proximal and distal colon tumors as distinct biologic entities with different prognoses. 2013 ASCO Annual Meeting Abstract. J. Clin. Oncol. 31(suppl):3526.

[26] Boisen, M. K. , J. S. Johansen , C. Dehlendorff , J. S. Larsen , K. Osterlind , J. Hansen , et al. 2013 Primary tumor location and bevacizumab effectiveness in patients with metastatic colorectal cancer. Ann. Oncol. 24:2554–2559. 23864097 10.1093/annonc/mdt253

[27] Volz, N. B. , S. Stintzing , W. Zhang , D. Yang , Y. Ning , T. Wakatsuki , et al. 2015 Genes involved in pericyte‐driven tumor maturation predict treatment benefit of first‐line FOLFIRI plus bevacizumab in patients with metastatic colorectal cancer. Pharmacogenomics J. 15:69–76. 25069475 10.1038/tpj.2014.40

